# Early Biomarkers of Periodontitis: New Challenges for a Personalized Medicine Approach

**DOI:** 10.3390/ijerph19010251

**Published:** 2021-12-27

**Authors:** Gaetano Isola

**Affiliations:** Department of General Surgery and Surgical-Medical Specialties, School of Dentistry, University of Catania, Via S. Sofia 78, 95124 Catania, Italy; gaetano.isola@unit.it; Tel.: +39-095-378-2453

Recently, the concept of precision medicine has attracted attention [1]. Precision medicine is a novel approach that predicts or diagnoses the disease before onset and comprises “treatments targeted to the needs of individual patients on the basis of genetic, biomarker, phenotypic or psychosocial characteristics that distinguish a given patient from the others”. One of the candidate biomarkers for precision medicine is microRNAs (miRNAs), which are short sequences, generally 19–25 nucleotides, that regulate the post-transcriptional silencing of targeted mRNAs, blocking translation or inducing the degradation of mRNA [2]. miRNAs are implicated in several physiological and pathological mechanisms and play important roles in inflammatory responses and the development of diseases, including cancer and rheumatoid arthritis [3]. miRNAs can be biomarkers for cancer diagnosis and prognosis, and specific serum miRNAs have been used as diagnostic tools for malignancies, autoimmune diseases, and infectious diseases [4].

Periodontitis is one of the most complex non-communicable diseases. Periodontitis progression results in periodontal attachment loss and/or tooth loss [5]. To prevent periodontitis progression, the realization of precision medicine for periodontitis is desired. Measuring salivary mediators—such as lactate dehydrogenase, lactoferrin, and 8-hydroxydeoxyguanosine—could be useful in diagnosing the periodontal condition [6,7]. Previous studies have reported that periodontal disease alters miRNA expression in human periodontal tissues [8,9,10]. Recently, specific serum and gingival crevicular fluid miRNAs were identified as biomarkers for chronic periodontitis [11]. However, the details remain unclear because these studies were cross-sectional, not longitudinal. A recent review suggested that a few salivary miRNAs have high diagnostic, prognostic, and therapeutic potential [11]. Nevertheless, it also suggested that further investigations are required, because there are not enough diagnostic and prognostic biomarkers of periodontal disease. Thus, we hypothesized that salivary miRNAs can be useful as biomarkers for predicting periodontitis progression. The aim of this cohort study was to identify miRNAs associated with periodontitis progression among chronic periodontitis patients receiving supportive periodontal therapy, which will contribute to predicting the onset and progression of periodontitis and achieving precision medicine in the field of oral health. More specifically, bacteria from the mouth can cause infection in other parts of the body when the immune system has been compromised by disease or medical treatments (e.g., infective endocarditis) [12,13]. Systemic conditions and their treatment are also known to have an impact on oral health (e.g., reduced saliva flow, altered balance of oral microorganisms) [14].

In this regard, saliva has been studied thoroughly as a potential diagnostic tool because its collection is non-invasive and economical. Salivary miRNA expression is modulated by various systemic diseases [15].

Advances in genetics and molecular biology have improved our knowledge of cellular mechanisms, which provide insights into the pathophysiological processes that turn healthy epithelial cells into cancer. Potential biomarkers and therapeutic targets can be investigated to identify genetic signatures that could be used for early diagnosis, treatment personalization, and, finally, the prognosis for individual patients.

In this regard, saliva has been described as ‘the defender of the oral cavity’ and provides for the protection of hard and soft tissues; aids taste, swallowing and digestion; and offers antimicrobial properties [16].

Saliva contains more than two thousand proteins, enzymes, electrolytes, small organic molecules and antimicrobials. The whole saliva contains plasma-derived components, sloughed epithelial cells, microorganisms and their associated products, gingival crevicular fluid, debris, and nasopharyngeal discharge. In the context of oral disease, the research, identification and use of salivary biomarkers is ongoing for many conditions [17,18].

In the Special Issue “New Biomarkers and Diagnostics in Oral Cancer and Oral Diseases”, there was an analysis of the recent advances in the field of oral diseases. More specifically, among published manuscripts, Mazurek-Mochol et al. [19] examined the association between the IL-17F rs763780 and IL-17A rs2275913 polymorphisms and periodontitis in non-smoking and smoking patients to check if these polymorphisms could be a risk factor for periodontitis. Interestingly, they found a lack of statistically significant associations between IL-17F rs763780 and IL-17A rs2275913 polymorphisms and periodontitis in a European population.

Recently, an increasing number of reports in the literature have focused on salivary research initiatives, the key objective of which is to generate a growing appreciation of the importance of saliva for overall health and for the diagnosis of oral diseases, and also to encourage a call to action in scientists and leaders in oral health to obtain the benefits of salivary screening tests useful for the advanced detection of oral disease that will help to stratify the patient’s risk and to reduce the global burden according to the “personalized medicine” approach especially in some conditions as malocclusions [20], dental transposition [21], and periodontitis [22]. 

This diagnostic modality in the field of molecular biology has led to the discovery of the potential of salivary biomarkers for the detection of oral cancers. Biomarkers are molecular signatures and indicators of normal biological and pathological processes, and pharmacological response to treatment, and hence may provide useful information for the detection, diagnosis, and prognosis of disease.

It is ever more apparent that addressing this challenge to improve oral health worldwide will require a closer and more robust engagement across sectors in the dental field and the adoption of an upstream approach to reduce the global burden of disease in general.
